# Guidelines for a Comprehensive Care Program to Ostomized Patients and Families: a Nursing proposal^1^

**DOI:** 10.1590/1518-8345.0507.2694

**Published:** 2016-05-17

**Authors:** Paula Alvarenga de Figueiredo, Neide Aparecida Titonelli Alvim

**Affiliations:** 2 PhD, RN, Conselho Regional de Enfermagem (COREN), Rio de Janeiro, RJ, Brazil. RN, Prefeitura Municipal de Campos dos Goytacazes, Rio de Janeiro, RJ, Brazil; 3 PhD, Associate Professor, Escola de Enfermagem Anna Nery, Universidade Federal do Rio de Janeiro, Rio de Janeiro, RJ, Brazil

**Keywords:** Nursing, Ostomy, Family, Needs Assessment, Integrality in Health

## Abstract

**Objectives:**

describe care needs and demands that mark the discursive practices of ostomized clients and family members and discuss guidelines for a comprehensive care program to ostomized clients and their families, organized by macrosociological categories.

**Method:**

Creative and Sensitive, involving 17 ostomized subjects and family members at a municipal outpatient clinic. The ethical aspects were complied with. A characterization form was used, as well as Creativity and Sensitivity Dynamics: "speaking map", "body-knowledge" and "calendar". Critical Discourse Analysis was applied.

**Results:**

the health needs and care demands of the ostomized patients and their family members, in their multiple dimensions, were constituted in the home and community, outpatient and social context, implying new orientations for nursing care. The unveiling of the data brought elements that constituted guidelines, in a macrosociological approach, to achieve the expanded integrality of nursing care.

**Conclusion:**

the ostomized clients are unique in their genre/peculiar from Latin sui generis, calling for strategies that respond to and distinguish their specificities. Elaborating a Public Health Policy that improves and reorganizes the care demands, taking into account these individual biopsychosocial and spiritual aspects, is a possible and irrevocable target in the attempt to achieve better conditions of health and wellbeing.

## Introduction

The guidelines for a comprehensive care program to ostomized patients and their family, proposed in this articles, are considered here as a process-based technology in a macrosociological approach. Social technology is understood as a set of applied arts and social techniques to support social work, serving as a tool of social inclusion and improvement of quality of life. Hence, it comprises a replicable product, technique or method, developed in the interaction with the community, and represents and effective solution of social transformation^(^ 1 ^)^. Adding up to the definition, the term process means reflexive activity intended to achieve knowledge on something. Thus, as part of the nurse's modes of action, based on the valuation of culture, it could characterize a clinical nursing care practice^(^ 2 ^)^.

In view of the social group studies, the stoma is a surgical derivation of viscera (generally intestine or urinary tracts) to the skin at a different point from the natural excretion orifice. In this surgery, the colon or part of the urinary system (generally bladder or urethra) is linked to the abdominal wall, communicating them with the outside with a view to the elimination of feces and/or urine. Thus, the stoma patients starts to use a collector bag stuck to the abdomen to protect the skin^(^ 3 ^)^.

The social repercussions in health for stoma patients and their family start when the stoma needs to be installed and there are difficulties to accept this new situation. There exists evidence that the diagnosis and installation of the stoma influence the lives of the patients and the people they relate with. The clients suffer a biological rupture and are confronted with different changes in their life process, ranging from the change in the gastrointestinal physiology, when they lose control of their sphincters, from self-esteem to altered body image, acknowledging their body as dysfunctional. Hence, the stoma alters not only the biological system, but also affects the individuals emotional and physiologically, impairing their social relationships. Therefore, they feel stigmatized as they suffer surgical complications in the postoperative phase and face problems in the experience of their sexuality. In function of the anatomical change of their body, the way of life of patients living with a stoma for elimination changes. These transformations, in turn, condition the patient's family, affect, professional and social life, entailing the need for family support and a professional care structure with a view to faster and more effective rehabilitation^(^ 4 ^)^.

Based on the observations in the Group of Ostomized Patients that served as the research scenario, it could be evidenced that, in daily nursing care, the support granted to ostomized subjects' problem, in its multiple dimensions, privileges the restoration and maintenance of the physical body damaged by the surgical derivation. This care model, which remains rooted in the biomedical perspective, does not highlight the multiple Social Determinants of Health (SDH) that strongly influence the course of life of stoma clients and their families, such as social, economical, cultural, ethnic/racial, psychological and behavioral factors that, as a whole, determine and condition the maintenance of health and the occurrence of diseases. In that perspective, the differences in health conditions among human groups may not be justified by biological factors but, on the opposite, the differences in health conditions seem to result from socially constructed habits and behaviors and, mainly, from factors beyond the individual or group's direct control^(^ 5 ^)^.

As social human beings, stoma patients and their relatives are subject to these SDH, linked to their life and health conditions, inherent in the family system with an elimination stoma. Therefore, they need to be unveiled with a view to investing in the "causes of the causes" of the health problems and their developments, facing the difficulties in life with the surgical derivation, entailing the possibility to achieve the integrality of care. The emergence of a specific program to consolidate the organization of a support network and the operation of a health care process for this group, in the singular space, should be considered. In that sense, the goal was: to describe the care needs and demands that mark that discursive practices of ostomized clients and their families, and to discuss guidelines for a comprehensive care program to ostomized clients and their family, organized by macrosociological categories.

Concerning the public policies for stoma patients, these have gradually advanced to support best practices in the Unified Health System (SUS). In search of alignment with international studies, the lack of research that directly focuses on health policies for the social group being assessed could be observed. One North American study on the Development of a Self-Management Program for Stoma Care is highlighted, which emerges from the stoma patients' quality of life recommendations/suggestions, considering the impact of the surgical derivation on physical, psychological, social and spiritual aspects^(^ 6 ^)^. Paying attention to the public policies in force in care to stoma clients and their families can serve as an important tool to assert the rights and duties of SUS users, managers and health professionals.

## Method

Qualitative field research developed through the Creative and Sensitive Method (CSM) as the data production axis for the construction, analysis and validation of the research results^(^ 7 ^)^. The discussion on the social relationships and their repercussions for health serves as the theoretical background to study the achievement of care integrality in view of the contemporary order of capital that alienates and thingifies the relations between the social being and the Social Determinants of Health in the model by Dahlgren and Whitehead^(^ 8 ^)^, coping with inequities in health at the different degrees of governability: the general, the private and the singular^(^ 9 ^)^, as well as the problematizing pedagogy in health, mediating the dialogue with stoma clients and their relatives^(^ 10 ^)^.

The participants were 11 ostomized clients, being seven adults and four elderly; and six family members, being four adults and two elderly, involved in and affected by the stoma problem in their daily life, totaling 17 subjects. The participants were distributed in four distinct groups of data production, consisting of different family systems, in view of the criterion that groups should contain between six and eight members at most. The participants were identified using alphanumerical codes, in the order in which the data were produced, described as follows: researcher/group coordinator (C), research aids (A) and ostomized patients (O). The family members were identified according to the number attributed to their ostomized patient (F1, F2...). Each group was also identified for the sake of a better organization of the data production and analysis: G1, G2, G3 and G4.

The research scenario was a Group of Ostomized Patients at a municipal outpatient clinic located in Campos dos Goytacazes, RJ. The project was registered on the *Plataforma Brasil*, in compliance with National Health Council Resolution 466/12, Ministry of Health, and approved on November 27^th^ 2012, Opinion 155.748, CAAE 07399512.2.0000.5238. To develop the research, the following steps were implemented: request for authorization to access the place of study, entry in the scenario and start of the data production process, based on the random collection of potential subjects, which took place at the place of study between December/2012 and March/2013, where individual interviews were held to collect the participants' characteristics, after they had accepted to participate in the research by signing the Informed Consent Form (ICF). In that sense, during other meetings, three types of Creativity and Sensitivity Dynamics (CSD) were developed with each group of participants, called "Spoken Map", "Body-Knowledge" and "Calendar", in that order.

The CSD "Spoken Map", rooted in the social space, was conducted as follows: on a sheet of paper, each participant (ostomized client and family member) should freely draw and/or write the places/routes taken to attend to their needs after the stoma, outlining limits and challenges they face along this route. The central theme of the CSD "Body-Knowledge" was "the care demands, what they need to take care and what they do", where each group participant manifested the place the maintenance care for the stoma client's body and the relative's body occupied on the silhouette of four physical bodies, two females and two males, drawn on a sheet of brown paper for the participants to frame. The CSD "Calendar" served to synthesize the data production: on a sheet of paper, the subjects should develop an individual artistic production linked to the central theme about what the ostomized client (or relative) needs to feel complete and taken care of. The participants were offered several magazines, scissors, glue, colored and ballpoint pens.

The group discussions were audio recorded electronically. Next, the artistic productions were organized and digitalized. The combination of the data developed with all the techniques made up the textual corpus of the research production report, subject to floating reading to apprehend the main ideas and apply the Critical Discourse Analysis (CDA), triangulating the data that emerged^(^ 11 ^)^.

## Results

Among the 17 research participants, 11 were ostomized, with a predominance of ostomized (7), colostomized (9) women. On average, the surgical derivation had been installed for 113 months. Ages ranged from young adults to elder elderly. The majority (6) was married, had finished secondary education (4), was evangelical (5) and Catholic (5) and gained a mean family income of one minimum wage (6). The six participating relatives had the following degree of family relationship: husband (2), cousin (1), aunt (1), sister (1) and wife (1). The majority (5) started the monitoring in the preoperative phase of the ostomy, were female (4), between 34 and 67 years of age. The marital situation of most of them was married (3), being (1) divorced and (2) single, with unfinished primary education (3), evangelical (3) and Catholic (3). A family income between one and three minimum wages was predominant (3).

The research participants used language as a form of social practice. The health needs, care demands and borderline situations, in their multiple dimensions, which marked their discursive practice, were constituted in the domestic and community, outpatient and social context, one influencing the other, implying new orientations for nursing care. The stoma clients had different causes underlying the installation of the stoma, which they called "surgery of life" (O7, O9, O11), being the *start of the whole new life* (O1). The causes of the adult and elderly, colostomized or ileostomized research subjects' permanent or temporary stoma included reports of tumors, polyps, Firearm Perforation (FP), ulcerative rectocolitis, Chron disease and medical errors, such as inappropriate diagnosis and intestinal abscess due to tubal infection, caused by an examination done wrongly.

The participating stoma patients highlighted that, to feel complete and truly taken care of, they need to *eat and accomplish dreams, cultivate hope and be optimistic about the future in view of the new condition of being ostomized* (fragment of synthesis by coordinator of CSD "Calendar" - G2; G4 and O8/G3), despite difficulties to accept it: ... *a good environment for our daily care, healthy eating, calm place... for you to take care of yourself... People's love, the kindness of our relatives...* (O4).

The needs and demands of the stoma patient's physical body include the needs and demands related to the maintenance of the stoma, with particular attention for the particularities of elderly stoma patients; basic hygiene and esthetic care: use of adjuvant and accessory equipment that attends to their specificity (drainable or closed collector bag, protective skin barrier in paste and power, deodorant lubricant, belt and fixing tape). The quality of the material offered in the market is a constant concern, as what best adapts to the user is not always considered, from the perspective of his wellbeing and comfort, but what corresponds to the interests of the economic system: ... *I had to buy the box of bag and stomahesive I bought it, because I left the hospital, look, I didn't do it through X, I did it through a private hospital...* (O11). *This bag, one possesses one quality and the other another, you see?* (F4). *It's the quality of the bag...* (F3). *... for us ostomized patients it would be good to have like... a place just for us, you see, mainly a bathroom...* (O4).

Care with meals also stands out because of some adaptations and dietary restrictions the clients go through because of the stoma. The knowledge gained from experience in this groups helps to test foods, identifying foods that help to neutralize odors or produce strong smells, foods that soften the feces or provoke intestinal constipation and others that produce increased flatulence. In addition, the location of the stoma, the baseline disease and comorbidities are factors inherent in the diet to be followed: *... then I also mentioned the good, because a good diet is extremely important always, despite our restrictions because of the stoma, right?!* (O11).

As regards the access to professionals and support networks, besides the family as an important core of support and care, the networks that take care of the body image (beauty salons) and spirit (church) stood out in their respective social spaces. Concerning the socio-interactive needs, the family constitution, leisure and work were emphasized. The latter serves as a form of social acknowledgement, besides constituting an escape line from different kinds of problems deriving from the presence of the stoma. The availability of technology, such as the mobile phone, also favors the social interaction: *... I am unable to sit still, work occupies the mind when you occupy the mind, often I even forget that I use the bag* (O1). *... contact with nature, contact with people and with the human being created by God...* (O4).

The need to have their rights guaranteed is also highlighted as a matter of citizenship: *... because in society the laws refer to the rights of citizens, and which should be respects, and because it's also difficult for us to get safety and health care...* (F1).

Some needs and demands of the family members are similar to those of the stoma patients. Besides basic care for health maintenance, the relatives exhibit dynamics characteristic of everyone living with people who present some kind of impairment or who truly serve as caregivers. In turn, they suffer the social consequences it implies. The thematic synthesis of the groups highlighted important aspects for the relatives' physical and psychospiritual health: waking up early, use of sunscreen, use of sunglasses and prescription glasses, going to the gym, exercising and stretching, healthy eating habits, water intake, sleep and rest, hygiene and bodily esthetics, creation and maintenance of bonds at work, with neighbors, friends and family, need for a healthy and calm mind and spiritual and religious support, such as going to church frequently, reading the bible and praying. In the inter-discourse produced, the care needs and demands inherent in the relatives' health deserve attention and inclusion in a nursing process, with a view to achieving the integrality of health care. The family system needs to be balanced to jointly cope with the limitations imposed on the wellbeing of ostomized patients and their family members.

In this social group's discursive practice, borderline situations also emerged as a frontier/problem/challenge for the family system's life and health dynamics, as follows: fragmented social networks, contradictions/conflicts in the search for a healthier lifestyle, general environmental conditions and unbalanced/maladjusted/disorganized housing conditions and their influence on self-care, unfavorable socioeconomic conditions, alienation from work and its consequences and demands for human life, lack of professional care or its instability, particularities of elderly stoma patients - vulnerability to repercussions of the stoma, the social environment of the stoma patient and its influence on the psychological aspect and how it promotes social practice. Particular attention should be paid to the difficulty to accept his physical condition in the social sphere, the production of foods and its influence on the behavior of the intestinal elimination stoma. These elements were taken into account in the planning of the macrosociological approach guidelines, with a view to better conditions of health and wellbeing, considering the need to achieve integral health care.

## Discussion

The integrality of health care needs to be addressed in different dimensions to be achieved as fully as possible in health care. Further approximation between professionals and users is needed, so that the needs of individuals and groups start to guide the actions, breaking with the vertical imposition of conducts^(^ 12 ^)^. In that perspective, care for a user is an effort towards a complete, holistic and therefore integral approach of each person with health needs who, for a certain period in his life needs nursing care.

These needs, characteristic of the human condition, as "needs for food, shelter, reproduction and health, besides autonomy and freedom" and the respective demands, "products of the social relations and linked to lacks" are social and historically determined^(^ 13 ^)^. They involve a process of measuring the health shortage in a population, in the form of losses in a certain health profile. Assessing them implies constructing a health or disease profile. The need grows as the health deficit increases.

Understanding that these needs can be captured in the microspace of care, from a macrosociological perspective, producing care demands in health and nursing, these demands need to guarantee, in their modus operandi, the possibility to see the client as a whole, in constant interaction with the dynamic environment, allowing the client to feel complete and truly taken care of. The elements of the macrosociological dimensions (biological, psychological-affective, esthetic, ethical, political, economic, social, professional, environmental, technological, cultural, educational and religious-spiritual) that permeate the life of human beings are fundamental to achieve health and wellbeing. All health needs and care demands included in the macrodimension need to be considered to plan actions each professional needs to perform with the users who need care. Therefore, a retun to the microspace of care is needed to support professional attitudes coherent with the specific health needs of each social group and each human being.

The abovementioned dimensions go beyond the health needs of the people without this kind of surgical derivation. The family system of the ostomized patients is gradually affected and their members are involved in the existential movement of these dimensions, assuming responsibilities to offer the support needed in the context of this new state of being a family. As a result of this imposed condition, the family gains new health needs and care demands that accompany the therapeutic itinerary of their ostomized relative, beyond what existed before the ostomy.

Considering health as the synergy of different factors and the subjects as a biopsychosocial and spiritual complex, the current public policies aim to welcome them in their context and take precedence through autonomy, physical and moral integrity. Therefore, the basic intent is to comply with the doctrinarian principles of the SUS regarding universal access to services, integrality and equality in health care. The National Humanization Policy is highlighted, concerning its emphasis on the social needs and interests of the different actors involved in health. Its political actions, therefore, are based on the challenges of constructing and expanding the spaces of exchange among these actors, in the advance from a new ethics that moves and sustains the institutional support function^(^ 14 ^)^. Thus, to devise the organization of a Comprehensive Health Care Program for Ostomized People and Families in the Outpatient Context, these subjects need to be valued as protagonists of care, sociocultural beings with specific demands, and situate them as active participants in the health care process. Therefore, it is important to invest in guidelines organized according to macrosociological dimensions, as discussed next.

User participation in health conferences and councils needs to be encouraged, developed in the context of the SUS, besides user satisfaction research with a view to improving the service offered/shared. Investments are needed in the integration of the outpatient clinic with the health service network, from the perspective of interdisciplinarity, adding other types of services that go beyond the traditional concept of health, in view of the need to work with the intersectoral approach in the contemporary world, guaranteeing referral and counter-referral.

Concerning the biological and psychological-affective dimension, the physical and socioemotional particularities of body maintenance of intestinal and urinary stoma users are highlighted: change of (drainable and closed) collector bad, maintenance of closed collector bag, daily maintenance of drainable collector bag for urostomy, self-irrigation, use of adjuvants (skin protection barrier in paste and powder, deodorant lubricant), prevention of late complications (dermatitis, prolapse and hernia), use of accessories (belt and adhesive tape), sun bath and equipment protection during common bath, with a view to reaching a healthy skin around the stoma, color, shape, size, protrusion, moisture, mucous integrity - with normal characteristics of a stoma, effluent control, pain relief, self-esteem, safety, autonomy, comfort, wellbeing, happiness, satisfactory sexual activity, self-concept, congruent daily activity, increased life expectancy, among others. The increased availability of collector bags and adjuvants that attend to the emerging singularities of clients in the SUS context should be highlighted.

In the esthetic dimension, as a form of physical human beauty, the supply of esthetic courses and treatment options for stoma patients should be taken into account, with a view to contributing to the maintenance of their self-esteem and well-being, mainly focused on women. In the socioeconomic and professional field, financial counseling in the family system is relevant, besides encouraging the effective participation of health professionals, stoma clients and their relatives in the Association of Ostomized People. The promotion of professional courses for ostomized people should be considered, with a view to facilitating their (re)insertion in the job market, paying attention to the limits and possibilities to assume certain functions that may diverge due to their health condition.

The need emerged to promote a healthy outpatient and home environment, covering the aspects of hygiene, temperature, ventilation, lighting, color therapy and aromatherapy. In the technological dimension, the personal needs related to SUS are considered, such as the need to promote technological participation. In addition, the professional needs are considered with a view to achieving a health work process with individual and collective care, aiming to maximize and control health care involving clients with a temporary elimination stoma and their relatives, concerning the care and support needed for reconstructive surgery; individual and collective care for the relatives of stoma patients, so as to include them in care for their ostomized relative and also to receive care; the implementation of consults by different professionals: nurses, physicians, social workers, psychologists and nutritionists, for support and adjustment of the disequilibria due to the confection of the stoma in the different dimensions of the lives of clients with intestinal/urinary stoma and their family members; identification and monitoring of late complications; implementation of home visits to understand the home context and family dynamics and dialogued orientations on self-care; besides the management of material, staff and physical structure, inherent in the functioning of the outpatient clinic and the development of activities promoted by health professionals working at the service and registered users.

In the cultural dimension, the development of playful and sociocultural activities is highlighted, such as games, sports, music, drama appropriate to the physical, social and mental capacity of each participant. Also, the educational field comprises: dialogical, group-based and individual education in health for the problematization of borderline situations, experienced in the concrete reality of the people involved in the care process deriving from the installation of the stoma, with a view to achieving self-care and permanent education for the health professionals involved. In addition, the development of interdisciplinary field studies is highlighted, in response to the problems that emerge in this social group and the dissemination of the knowledge produced. The religious-spiritual need strongly emerged in the statements of the ostomized participants and their relatives, evidencing the health care demand that guarantees respect for these subjects' religiosity and/or spirituality.

When considering the implementation of these guidelines, organized according to the macrosociological dimensions mentioned, the interdisciplinary perspective of health needs to be adopted. This is a common thematic point that acts as an integrator and mediator in the circulation of disciplinary discourse, in which reciprocity, mutual enrichment and a trend towards the horizontalization of the power relations between the fields coexist, overcoming the disciplinarity and fragmentation of knowledge and therefore relating it with the reality and the problems of modern life, in search of care for the integrality of human beings^(^ 15 ^)^.

In the field of interdisciplinarity, the team's efforts will be directed at the situation inherent to each health service. Different professionals with a particular/specific background will adopt distinct perspectives in function of a common objective, which does not mean the juxtaposition of knowledge, nor the annulment of the specificity of each field. This nevertheless implies awareness of the limits and potentialities of each knowledge field with a view to opening towards collective action^(^ 16 ^)^.

With a view to the appropriate functioning of a Comprehensive Health Care Program for Ostomized People and Families, a physical structure should be provided that is relevant to the activities to be developed, with qualified and quantitatively sufficient staff, according to the number of registered users, printed material and/or the computerization of health care, collectors and protective and safety equipment for stoma patients, as well as fixed and daily consumption material for professional actions. It is important to invest in communication media like mobile phones and the internet, in view of their role in a globalized world where information is multiplied exponentially. The health professionals need to recover for the sake of continuing changes in daily practice, encouraging evidence-based practice and scientific research. This technology is also relevant for communication among health care networks, with a view to taking responsibility for the users' referral and counter-referral and for administrative activities.

Concerning the nurses' specific actions in the program, the nursing consultation is highlighted, involving the implementation of the five phases of the nursing process and continuing education, including training and educational programs. In professional nursing practice, the nurses are considered essential in the rehabilitation process of ostomized patients, as they are present since the diagnosis of the stoma^(^ 17 ^)^. In that interval, the nurses advise the patients about care for the stoma, meals, hygiene, preparing them for self-care and the return to daily activities.

It is important to consider the particularities of elderly patients concerning the changes characteristic of the aging process: anatomic-physiological, cognitive, functional and psychosocial^(^ 18 ^)^. This precaution will influence the organization of the stomal therapy service, the planning and implementation of care that will guarantee the specificities required to manage this social group's quality of life.

The nurses are responsible for devising health action strategies that transcend the disease focus, with a view to promoting means to help the elimination stoma users to make decisions, express feelings and help them to cope with the changes in their body image for the sake of survival^(^ 19 ^)^. In line with Brazilian studies, international research has identified coping experiences of stoma patients in the course of their lives, as physical, psychological, social and spiritual aspects are affected. Hence, they need adjustments to maintain their quality of life^(^ 6 ^)^, which is a possible target with the help of health services and nurses, whether specialists or not, despite the presence of the stoma^(^ 20 ^)^.

## Conclusion

The operation of health care, the commodification of care and the lack of effective application of basic concepts in the execution of humanized, comprehensive, technical work that is faithful to the SUS principles negatively affect the problem-solving ability of the borderline situations the people experience in the context they live in. Thus, investments are needed in the organization of a health service network that maintains a flow in line with the peculiar needs of a certain social group, with a view to achieving health in a broad sense. Achieving this comprehensive state of health, often considered utopic and revolutionary, which is dynamic in the course of daily life, represents an emerging challenge not only for the SUS users, but also for the health professionals and their managers, wherever they are. Nevertheless, it is not considered a naïve objective, but feasible when practicing and devising health from a macrosociological perspective, with a distinguished professional attitude, based on sensitive listening to the users' health needs and promotion of articulation and effective integration with the different spaces: general, particular and singular.

In the development context of this research in health and nursing, challenges and limits emerged in the conception phase of a singular project, and mainly in the data production phase, where difficulties occurred to capture the subjects and maintain the groups constituted to develop the CSD, although the reliability criteria were complied with, related to the saturation of the data: empirical, theoretical and categorical. The results discussed here are not enclosed in their construction, in view of the concept of knowledge as inexhaustible, unfinished and incomplete, besides the complexity deriving from the multiple changes the elimination ostomy causes in the lives of the people involved. Through these results, it was verified that the health needs of stoma patients and their relatives, as well as the borderline situations this social group is confronted with, are captured in the microspace of their social relationship, producing care demands in health and nursing. Imprinting a macrosociological perspective on these demands, based on the subjects' multiple voices in their singular space, through the professional's sensitive and qualified listening, is the route to achieve the expanded integrality of care.
